# Superior Mesenteric Artery Syndrome: A Potentially Fatal but Reversible Gastrointestinal Manifestation of Systemic Sclerosis

**DOI:** 10.1155/2020/8831417

**Published:** 2020-07-03

**Authors:** Choon-Guan Chua, Gervais Khin-Lin Wansaicheong, Wee-Chian Lim, Bernard Yu-Hor Thong

**Affiliations:** ^1^Department of Rheumatology, Allergy and Immunology, Tan Tock Seng Hospital, 11 Jalan Tan Tock Seng, Singapore; ^2^Department of Diagnostic Radiology, Tan Tock Seng Hospital, 11 Jalan Tan Tock Seng, Singapore; ^3^Department of Gastroenterology and Hepatology, Tan Tock Seng Hospital, 11 Jalan Tan Tock Seng, Singapore

## Abstract

Superior mesenteric artery syndrome (SMAS) is a rare gastrointestinal disorder characterised by vascular compression of the third part of the duodenum, in the angle between the superior mesenteric artery (SMA) and the abdominal aorta. It presents as an uncommon cause of upper gastrointestinal obstruction. In patients with systemic sclerosis (SSc), gastrointestinal involvement may result in oesophageal dysmotility, gastroesophageal reflux disease (GERD), gastroparesis, small intestinal bacterial overgrowth (SIBO), chronic intestinal pseudoobstruction (CIPO), and fecal incontinence. Malnutrition may thus result in weight loss and reduced mesenteric and retroperitoneal adipose tissue, decreasing the angle between the SMA and aorta causing SMAS. Enteral or parenteral feeding can potentially reverse SMAS in SSc. We report a case of SMAS in an elderly female with SSc and concurrent gastrointestinal involvement, and discuss the important management considerations and potential adverse outcomes when untreated.

## 1. Introduction

Systemic sclerosis (SSc) is a systemic autoimmune rheumatic disease where gastrointestinal involvement occurs in more than 90% of patients, more often in diffuse cutaneous SSc (dcSSc) rather than the limited forms. It is characterised by three key pathophysiological processes: inflammation, vasculopathy, and fibrosis. The four key pathophysiological mechanisms causing gastrointestinal dysfunction in patients with SSc appear to be as follows: infiltration of immune cells (via humoral and cell-mediated immunity) into gut smooth muscle; fibrosis of gut smooth muscle; labile vascular tone of the submucosal arterioles and venules; and dysfunction of the enteric nervous system (ENS) and smooth muscle [1]. It has recently been proposed that gastrointestinal dysfunction in SSc may be a staged process beginning with neuropathy and progressing to myopathy with eventual fibrosis [2]. Changes in the gut microbiota have also been demonstrated in contrast to healthy controls [3].

Gastrointestinal involvement may present with dysphagia, heartburn and regurgitation, nausea/vomiting, abdominal pain/distension, weight loss, constipation/diarrhoea, and fecal incontinence. Gastrointestinal involvement may result in oesophageal dysmotility, gastroesophageal reflux disease (GERD), gastroparesis, small intestinal bacterial overgrowth (SIBO), chronic intestinal pseudoobstruction (CIPO), and fecal incontinence [1, 2]. There has been little evidence to date of any role of immunosuppressive therapy for gastrointestinal disease in SSc, and prognosis remains poor [4]. Symptomatic treatment remains the mainstay of treatment, with enteral and parenteral nutrition being the key when malnutrition sets in [5].

Superior mesenteric artery syndrome (SMAS) is a rare gastrointestinal disorder characterised by vascular compression of the third part of the duodenum, in the angle between the superior mesenteric artery (SMA) and the abdominal aorta. This is most often a result of significant weight loss and reduced mesenteric and retroperitoneal adipose tissue, decreasing the usual angle of 38–65 degrees between the SMA and aorta, to less than 25 degrees causing SMAS [6].

## 2. Case Report

A 64-year-old Chinese female was diagnosed with dcSSc in 2011 at the age of 59 when she presented with diffuse skin thickening, Raynaud's phenomenon, and arthritis of the small joints of both hands. She subsequently developed progressive oesophageal dysmotility with absent peristalsis on oesophageal manometry. She did not have any small or large bowel involvement, interstitial lung disease, or pulmonary hypertension then. Antinuclear antibody (ANA) tested positive 1 : 160, centromere pattern; anti-Scl 70 and ribonucleoprotein (RNP) antibodies were negative. She was initially treated with methotrexate up to 10 mg/week. Her weight in June 2012 was 51.6 kg. Oesophagogastroduodenoscopy (OGD) in October 2012 showed *Helicobacter pylori*-associated active chronic gastritis and mild chronic oesophagitis. There were no histological features of Barrett's oesophagus. She declined OGD for surveillance for Barrett's oesophagitis, and colonoscopy between 2012 and 2016 despite progressive weight loss. Her weight was 42 kg by 2014.

She received methotrexate up to a dose of 10 mg/week till November 2015 for dcSSc and arthritis. This was then interrupted due to a prolonged hospitalisation for severe community-acquired pneumonia, type 2 myocardial infarction, critical illness myopathy, and polyneuropathy. In view of severe pharyngeal dysphagia, she was started on nasogastric tube (NGT) feeding from March 2016. Methotrexate was restarted in April 2016 but was discontinued in June 2016 when she developed infected left big toe gangrene. Other medical conditions that had developed during her disease course included hypertension, hyperlipidemia, and osteoporosis, for which yearly intravenous zoledronic acid was started in 2014.

**Figure 1:**
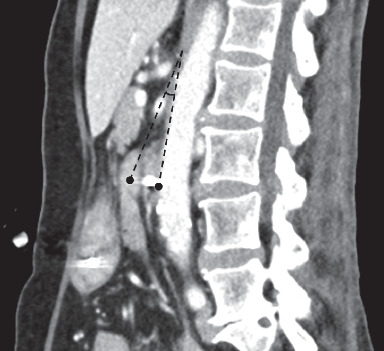
Sagittal multiplanar reconstructed CT scan image of the aortomesenteric angle (black dashed line) of approximately 23 degrees.

**Figure 2:**
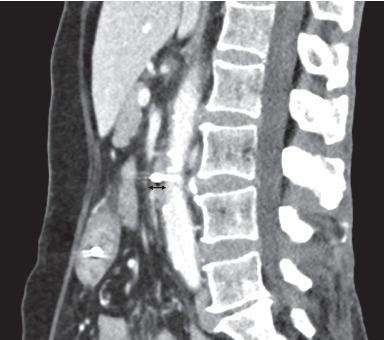
Sagittal multiplanar reconstructed CT scan image of the aortomesenteric distance (black line) of approximately 7 mm.

By July 2016, her weight was 41.5 kg. Her Hb was 10.6 g/dL (reference interval 11.5–16.5), and serum albumin was 27 g/L (35–50). Renal panel, serum calcium, phosphate, vitamin B12, and folate levels were normal. In August 2016, she was hospitalised for progressively worsening nonbilious nonbloody vomiting with high NGT aspirates and recurrent aspiration pneumonia. Her weight by then was 40.1 kg and height was 1.45 m (BMI 19.07 kg/m^2^). It was thus decided to insert a nasojejunal tube (NJT) under fluoroscopic guidance through the preexisting NGT for feeding. The stomach and proximal duodenum were grossly dilated with fluid. A transition point was encountered at the mid third part of the duodenum (D3) level with a vertically oriented obstruction suggestive of extrinsic compression by the SMA. Computed tomography of the abdomen-pelvis (CTAP) with intravenous contrast three days after the NJT insertion showed a narrowed aortomesenteric angle of approximately 23 degrees (Figure 1) and an aortomesenteric distance of approximately 7 mm (Figure 2). This was suggestive of SMAS. The stomach and duodenum were by then not distended, and the rest of the bowel was normal. Subsequent OGD showed corpus and antral gastritis with no intraluminal lesion. An NGT was left in situ for gastric suctioning and decompression. Total parenteral nutrition (TPN) was explored as an option to improve her nutrition in view of her progressive gastrointestinal involvement. The patient and her family were not keen in view of the long-term costs and potential complications for home TPN.

For the next three months, she was readmitted for recurrent pneumonia, small intestinal bacterial overgrowth, and repeated NGT/NJT blockage, which necessitated insertion of a double lumen Kangaroo NJ feeding/gastric decompression tube, which could be changed every three months. She passed away in November 2016 following another hospitalisation for pneumonia and acute myocardial infarction.

## 3. Discussion

Gastrointestinal involvement in SSc is associated with significant morbidity, mortality, and impact on health-related quality of life [7]. In a well-characterised local multiethnic systemic sclerosis cohort Singapore (SCORE) where patients fulfilled the American College of Rheumatology/European League Against Rheumatism (ACR/EULAR) or Very Early Diagnosis of Systemic Sclerosis (VEDOSS) criteria, 57% of deaths were attributed to SSc, with pulmonary arterial hypertension (PAH), interstitial lung disease (ILD), and gastrointestinal (GI) complications as the leading causes [8].

The manifestations of SSc patients with gastrointestinal involvement depend on the anatomical region affected and can be summarised as shown in Table 1. Approximately 50% of SSc patients experience small bowel involvement. The clinical presentation includes symptoms of chronic intestinal pseudoobstruction (CIPO) [9], bacterial overgrowth [10], and malnutrition [11]. These clinical manifestations are the result of reduced peristalsis with resulting bowel dilatation and stasis. CIPO presents as recurrent signs and symptoms of obstruction in the absence of a true mechanical obstruction where endoscopic features of a “hide-bound” mucosal surface may be seen [12]. Rare cases of megaduodenum have also been reported [13]. Patients with small intestine bacterial overgrowth (SIBO) can present with symptoms of abdominal pain, bloating, flatulence, diarrhoea, and weight loss. In severe cases, nutrient malabsorption may occur. Pneumatosis cystoides intestinalis (PCI) is a rare manifestation of small bowel involvement [14].

SMAS, also known as “Wilkie's syndrome” or arteriomesenteric duodenal compression syndrome, is characterised by the vascular compression of the third part of the duodenum, in the angle between the SMA and aorta, presenting with features of upper gastrointestinal obstruction. The causes of SMAS include severe body mass loss, metabolic conditions that reduces mesenteric and retroperitoneal adipose tissue, trauma, high anchoring of ligament of Treitz, low anchoring of the mesenteric artery, high-degree lumbar lordosis, neoplastic mass near SMA radix, dissecting aortic aneurysm, and some surgeries (e.g., surgical correction of scoliosis) [15]. The authors searched the Cochrane Library, PubMed, Embase, and MEDLINE databases from conception through 31 July 2017. The following search terms were used: “superior mesenteric artery syndrome,” “Wilkie's syndrome,” “arteriomesenteric duodenal compression syndrome,” “systemic sclerosis,” “scleroderma,” “connective tissue disease,” and “gastrointestinal manifestation.” No language restriction was imposed on the search. Case series, case reports, and the reference lists of articles were identified by this search strategy. Review articles were cited to provide readers with more details and more references beyond the scope of this case report.

Several nonrheumatological cases have been reported in the local literature, of whom all were adolescents [16, 17] or elderly above 60 years of age [18, 19]. One was in adolescent five weeks after scoliosis correction surgery [20]; the rest did not have an obvious cause for SMAS.

The clinical presentation of SMAS depends on the underlying etiology and degree of duodenum compression. They may exhibit signs and symptoms of proximal intestinal obstruction that include nausea, epigastric pain with early satiety, vomiting with partially digested food, and bilious contents. At times, resting in a lateral position helps alleviate symptoms by relieving the compression of the SMA on the duodenum. Depending on the severity and duration of disease, patients may develop dehydration, electrolyte imbalances, and weight loss. Physical examination yields nonspecific findings of abdominal tenderness and high frequency intestinal sounds.

The diagnosis of SMAS may be difficult and delayed due to nonspecific symptoms and physical examination findings from incomplete intestinal obstruction. It is a diagnosis of exclusion. Conventional angiography was considered in the past as the gold standard for diagnosis of SMAS. More recently, CTAP has been used as the reference standard for SMAS diagnosis as it is nonspecific and less expensive. Moreover, it can clearly demonstrate the aortomesenteric angle and distance accurately, as well as evaluate for other local pathologies (e.g., aneurysm and neoplasm) that could have led to the symptoms [21]. CTAP also allows the estimation of intraabdominal and retroperitoneal fat, in which other modalities such as ultrasonography and barium study do not provide.

SMAS-like manifestation was first described in systemic rheumatic disease in 42 cases of SSc in 1978. In this case series, all patients with duodenal dilatation had a compression defect at the site where the SMA crossed the duodenum. It was described that the compression defect and duodenal dilatation may spontaneously disappear and recur. However, the author attributed the compression produced by the SMA to dilatation and loss of muscle tone of the duodenum instead and did not accord a diagnosis of SMAS to the cases [22].

Definite SMAS has been described in a 32-year-old female with newly diagnosed systemic lupus erythematosus (SLE) presenting with serositis, lupus enteritis, nephritis, and cerebritis [23]. She developed SMAS following significant weight loss of 10 kg over three months at initial presentation. SMAS resolved with fasting and gastrointestinal decompression with an NGT, together with TPN for three weeks followed by cyclic parenteral nutrition. Repeat CTAP showed that the SMA-aortic angle, aortomesenteric distance, and fat volume estimation increased to 36.9 degrees, 3.4 mm, and 6082.460 cm^3^, respectively, upon recovery.

SMAS has also been described in a 35-year-old female with rheumatoid arthritis who developed cachexia following a two-year history of persistent nausea and vomiting after meals, with a weight loss of 12 kg in a year [24]. There was CT reduction of the aortomesenteric distance to 6.3 mm and aortomesenteric angle of 17 degrees with paucity of abdominal fat. This resolved with small frequent meals containing high calories and protein that resulted in a 5 kg weight gain over six months. She was restarted on methotrexate and sulfasalazine without adverse reaction. There are no reports of SMAS among the systemic vasculitides or other systemic rheumatic diseases. However, duodenal compression by the SMA against the abdominal aorta in thin children with Henoch–Schonlein purpura may lead to duodenal bowel abnormalities with abdominal pain that mimic SMAS [25].  Table 2 gives the summary of case reports and series on SMAS in patients with rheumatological conditions.

The potential anatomical defect in SMAS even if reversed with improvement in nutrition may not completely compensate for basal SMA vasoconstriction in SSc [26]. The latter may continue to impair absorption and aggravate the malabsorption due to small bowel wall fibrosis.

Management of SMAS comprises both medical and surgical approaches with the aim of reversing weight loss, thereby promoting restoration of retroperitoneal fat and increasing the aortomesenteric angle. Medical management includes intravenous fluid for rehydration correcting electrolyte imbalances; insertion of nasogastric tube for gastric and duodenal decompression, a nasojejunal tube insertion for feeding, frequent feeds with pureed and blended food, posturing manoeuvres to relieve obstruction, e.g., lying prone, left lateral, or in knee-chest position, use of prokinetic drugs such as metoclopramide or cisapride, and total parenteral nutrition. Surgical management that have been described includes laparoscopic duodenojejunostomy in children [27] and adults [28], Strong's operation [29], sectioning of the peritoneum anteriorly and superiorly between the duodenum and the pancreas and posteriorly and inferiorly between the duodenum and the posterior peritoneum, as well as sectioning the ligament of Treitz to free the duodenum with more caudal displacement, anterior transposition of third part of the duodenum [30], Billroth II gastrectomy, and transposition of the SMA to the intrarenal aorta.

Delayed diagnosis or persistent SMAS is associated with significant morbidity and risk of mortality. Severe hypovolemia, oliguria, hypokalaemia, metabolic alkalosis and other electrolyte abnormalities, aspiration pneumonia, oesophageal tear and upper gastrointestinal bleed, peptic ulcer and gastric perforation, cardiovascular collapse, and death [31] are potential consequences.

This patient unfortunately continued to develop recurrent blockages of her NGT and NJT. NGT feeding is often inadequate as food pools in the stomach and duodenum, proximal to the level of obstruction. Although NJ feeding have been effective in most cases [32], it would not have been effective in our patient given the poor absorption from scleroderma gut. Attempts to correct her weight to restore the retroperitoneal fat and increase the aortomesenteric angle were barely possible without TPN given the extensive SSc gut involvement.

## 4. Conclusion

SMAS is a potentially reversible gastrointestinal manifestation of SSc. Early diagnosis and definitive treatment in the form of enteral or parenteral nutrition may reverse malnutrition and restore the retroperitoneal fat pad. Delayed diagnosis or persistent SMAS is associated with significant morbidity and risk of mortality.

## Figures and Tables

**Table 1:** Manifestations of gastrointestinal tract involvement in systemic sclerosis.

Anatomical region	Manifestation
Oesophagus	Dysmotility Reflux oesophagitis Gastroesophageal reflux disease Stricture Barrett's oesophagus, adenocarcinoma (advanced)

Stomach	Gastritis and gastric ulcers Gastroparesis Gastric antral vascular ectasia (GAVE)

Small intestine	Hypomotility Pseudoobstruction Jejunal diverticuli Small intestine bacterial overgrowth (SIBO) Pneumatosis cystoides intestinalis (PCI)

Colon	Hypomotility Wide mouthed diverticuli Chronic intestinal pseudoobstruction (CIPO) Megacolon

Anorectum	Fecal incontinence

**Table 2:** Summary of case reports and series on superior mesenteric artery syndrome in patients with rheumatological conditions.

Author	Title	Publication	Description
Gondos [22]	Duodenal compression defect and the “superior mesenteric artery syndrome (SMAS)”	*Radiology* 123(3): 575–580	SMAS-like manifestation described in 42 cases of systemic sclerosis in 1978. In this case series, all patients with duodenal dilatation had a compression defect at the site where the SMA crossed the duodenum. It was described that the compression defect and duodenal dilatation may spontaneously disappear and recur. However, the author attributed the compression produced by the SMA to dilatation and loss of muscle tone of the duodenum instead and did not accord a diagnosis of SMAS to the cases.

Bedaiwi et al. [23]	Superior mesenteric artery syndrome and intraabdominal compartment syndrome in systemic lupus erythematosus	*Lupus* 23(2): 194–196	SMAS was described in a 32-year-old female with newly diagnosed systemic lupus erythematosus (SLE) presenting with serositis, lupus enteritis, nephritis, and cerebritis. She developed SMAS following a significant weight loss of 10 kg over three months at the initial presentation. SMAS resolved with fasting and gastrointestinal decompression with an NGT, together with total parenteral nutrition (TPN) for three weeks followed by cyclic parenteral nutrition. Repeat CT abdomen-pelvis showed that the SMA-aortic angle, aortomesenteric, and fat volume estimation increased to 36.9 degrees, 3.4 mm, and 6082.460 cm^3^, respectively, upon recovery.

Padhan et al. [24]	Superior mesenteric artery syndrome in an adult rheumatoid arthritis patient	*International Journal of Rheumatic Diseases* 15(1):e4-5	SMAS was described in an adult with rheumatoid arthritis who developed cachexia following a two-year history of persistent nausea and vomiting after meals, with a weight loss of 12 kg in a year. There was CT reduction of the aortomesenteric distance to 6.3 mm and aortomesenteric angle of 17 degrees with paucity of abdominal fat. This resolved with small frequent meals containing high calories and protein that resulted in a 5 kg weight gain over six months.

Chua et al.	Superior mesenteric artery syndrome: a potentially fatal but reversible gastrointestinal manifestation of systemic sclerosis	Current article	SMAS was described in an adult with diffuse cutaneous systemic sclerosis who developed recurrent nonbilious, nonbloody vomiting with high nasogastric tube aspirates and aspiration pneumonia. She lost about 10 kg over four years. During fluoroscopic guidance for nasojejunal tube insertion, the stomach and proximal duodenum were noted to be grossly dilated with fluid. A transition point was encountered at the mid third part of the duodenum with a vertically oriented obstruction suggestive of extrinsic compression by the SMA. Subsequent CT abdo-pelvis revealed narrowed aortomesenteric angle of approximately 23 degrees (Figure 1) and an aortomesenteric distance of approximately 7 mm. Patient declined TPN and eventually demised from acute myocardial infarction and pneumonia.
